# Supplementary figures and images for: Lineage priming and cell type proportioning depends on the interplay between stochastic and deterministic factors
Source: eLife. 2026 May 19;14:RP105512. doi: 10.7554/eLife.105512 (PMC13186565; doi:10.7554/eLife.105512)

AX4 set1<sup>-</sup> ash2<sup>-</sup>

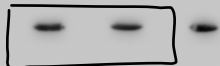

Supplement: Figure 5—source data 1. [file elife-105512-fig5-data1.zip › Figure 5 -source data 1/ax4_Set1_ash2_actin_10_secs.pdf]

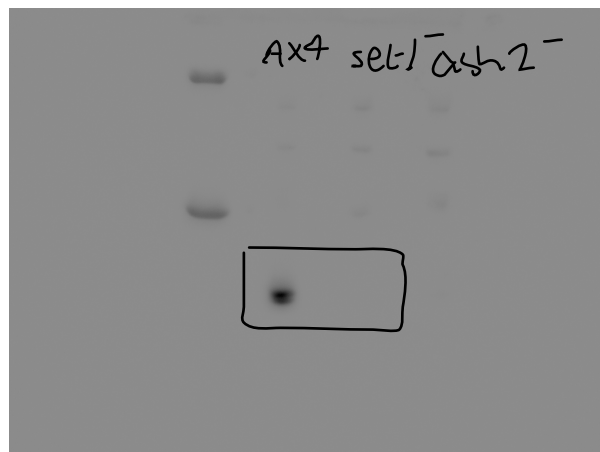

Supplement: Figure 5—source data 1. [file elife-105512-fig5-data1.zip › Figure 5 -source data 1/ax4_Set1_ash2_me3_10_secs.pdf]

Ax4 set1- ash 2-

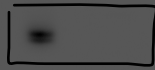

Supplement: Figure 5—source data 1. [file elife-105512-fig5-data1.zip › Figure 5 -source data 1/ax4_Set1_ash2_me2_10_secs.pdf]

AX9 setl<sup>-</sup> ash2<sup>-</sup>

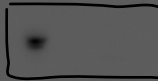

Supplement: Figure 5—source data 1. [file elife-105512-fig5-data1.zip › Figure 5 -source data 1/ax4_Set1_ash2_me1_10_secs.pdf]

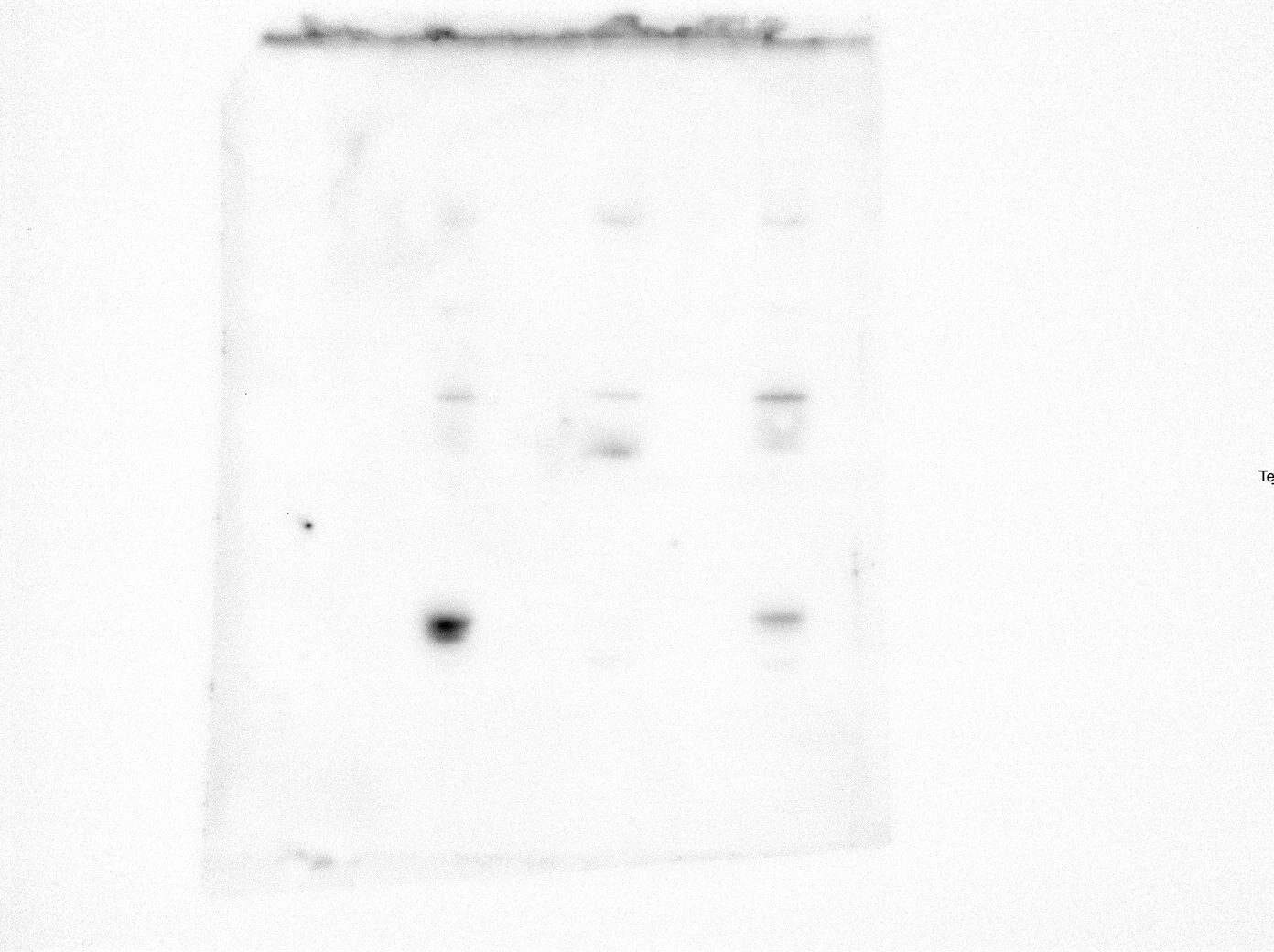

Supplement: Figure 5—source data 2. [file elife-105512-fig5-data2.zip › Figure 5 -source data 2/ax4_Set1_ash2_me1_10_secs copy.tif]

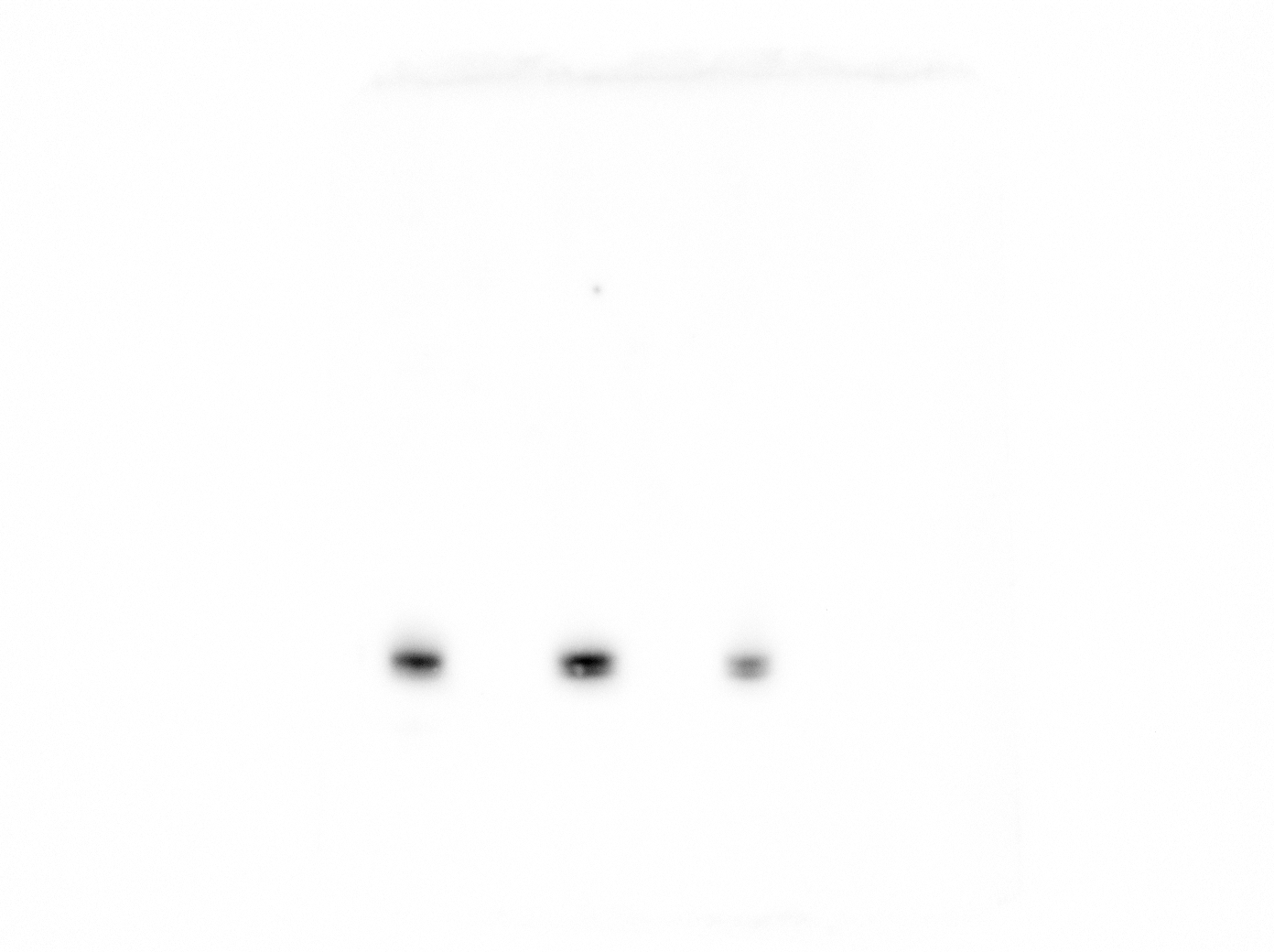

Supplement: Figure 5—source data 2. [file elife-105512-fig5-data2.zip › Figure 5 -source data 2/ax4_Set1_ash2_H3_10_secs copy.tif]

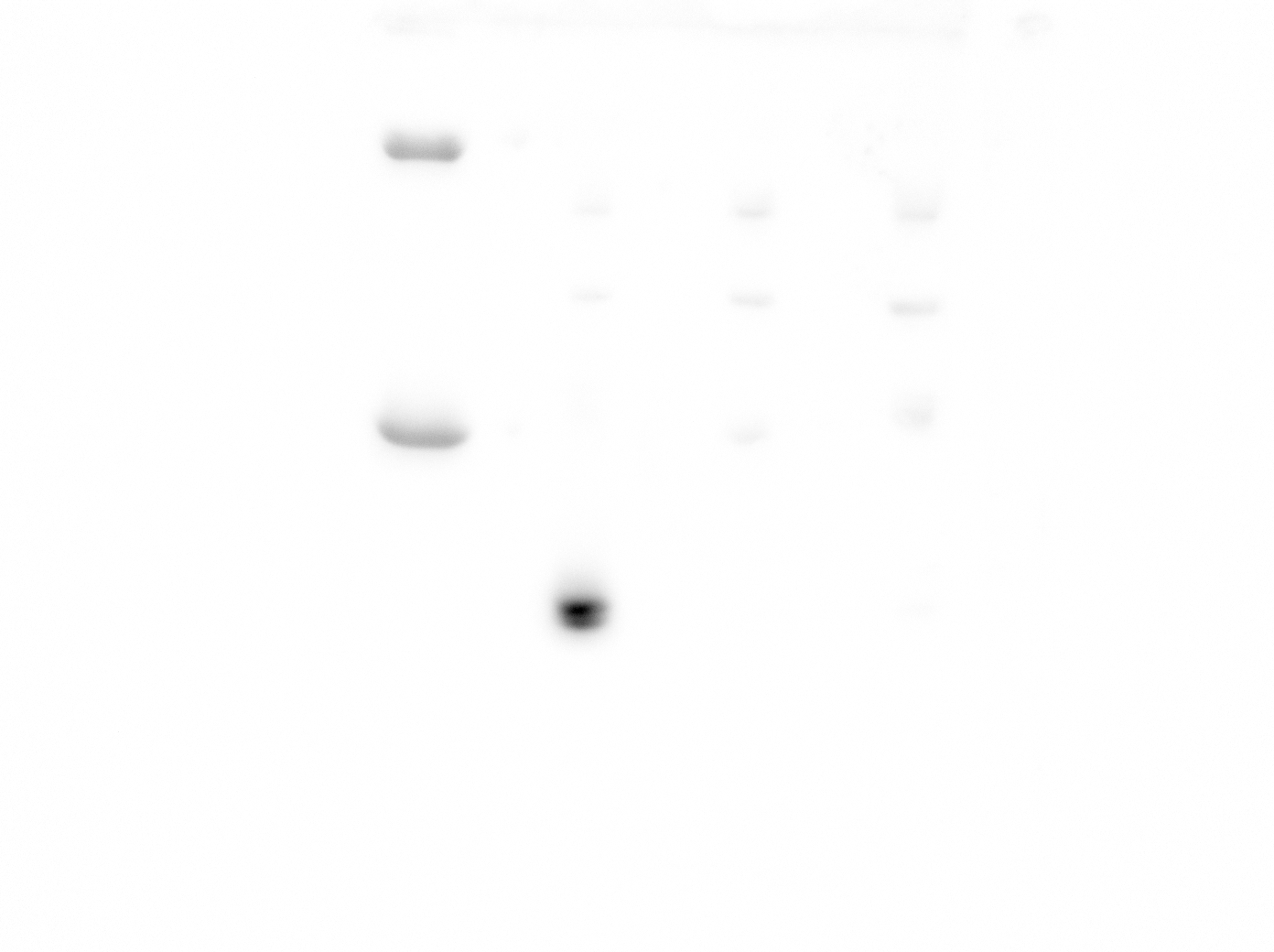

Supplement: Figure 5—source data 2. [file elife-105512-fig5-data2.zip › Figure 5 -source data 2/ax4_Set1_ash2_me3_10_secs copy.tif]

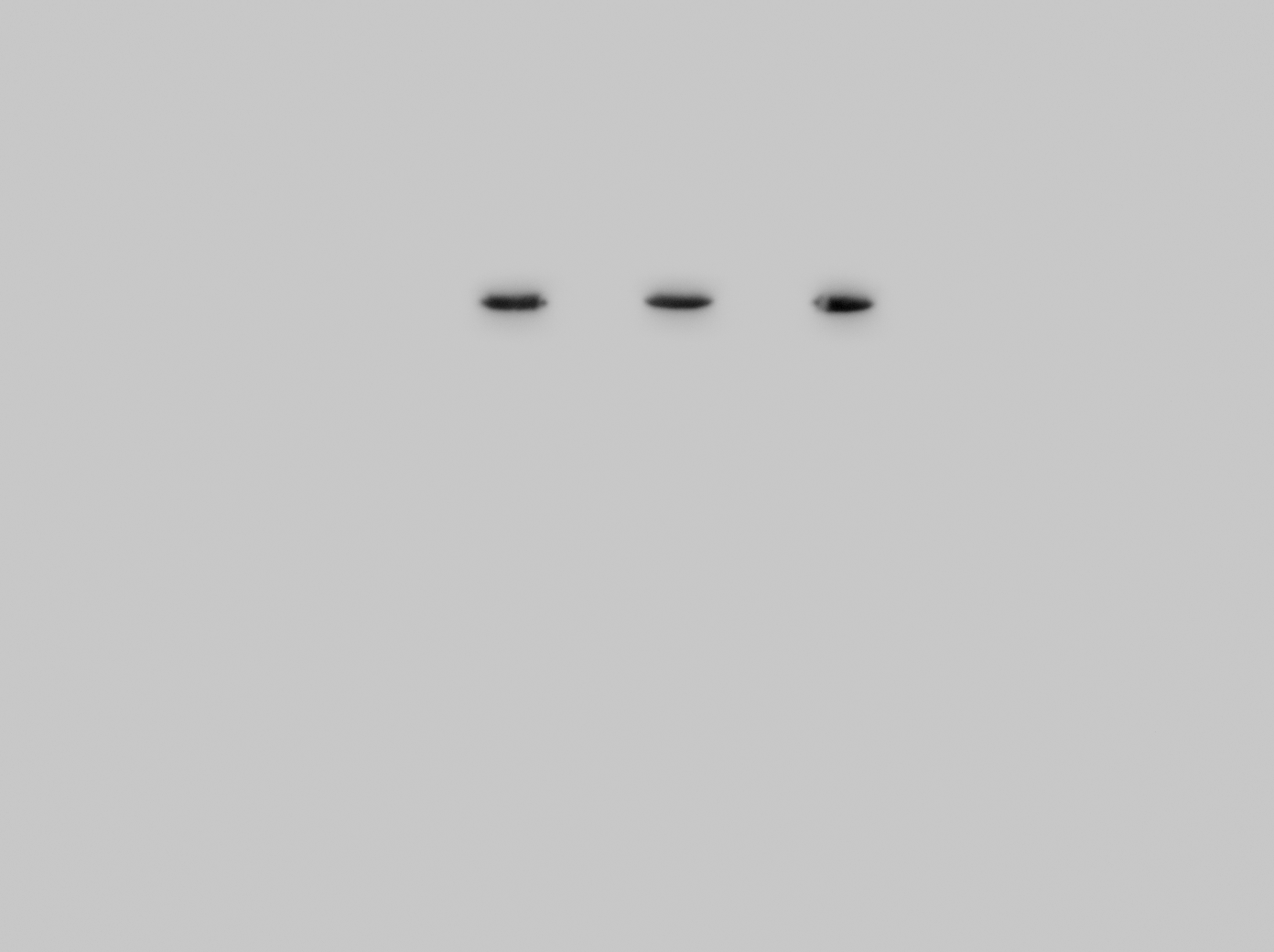

Supplement: Figure 5—source data 2. [file elife-105512-fig5-data2.zip › Figure 5 -source data 2/ax4_Set1_ash2_actin_10_secs copy.tif]

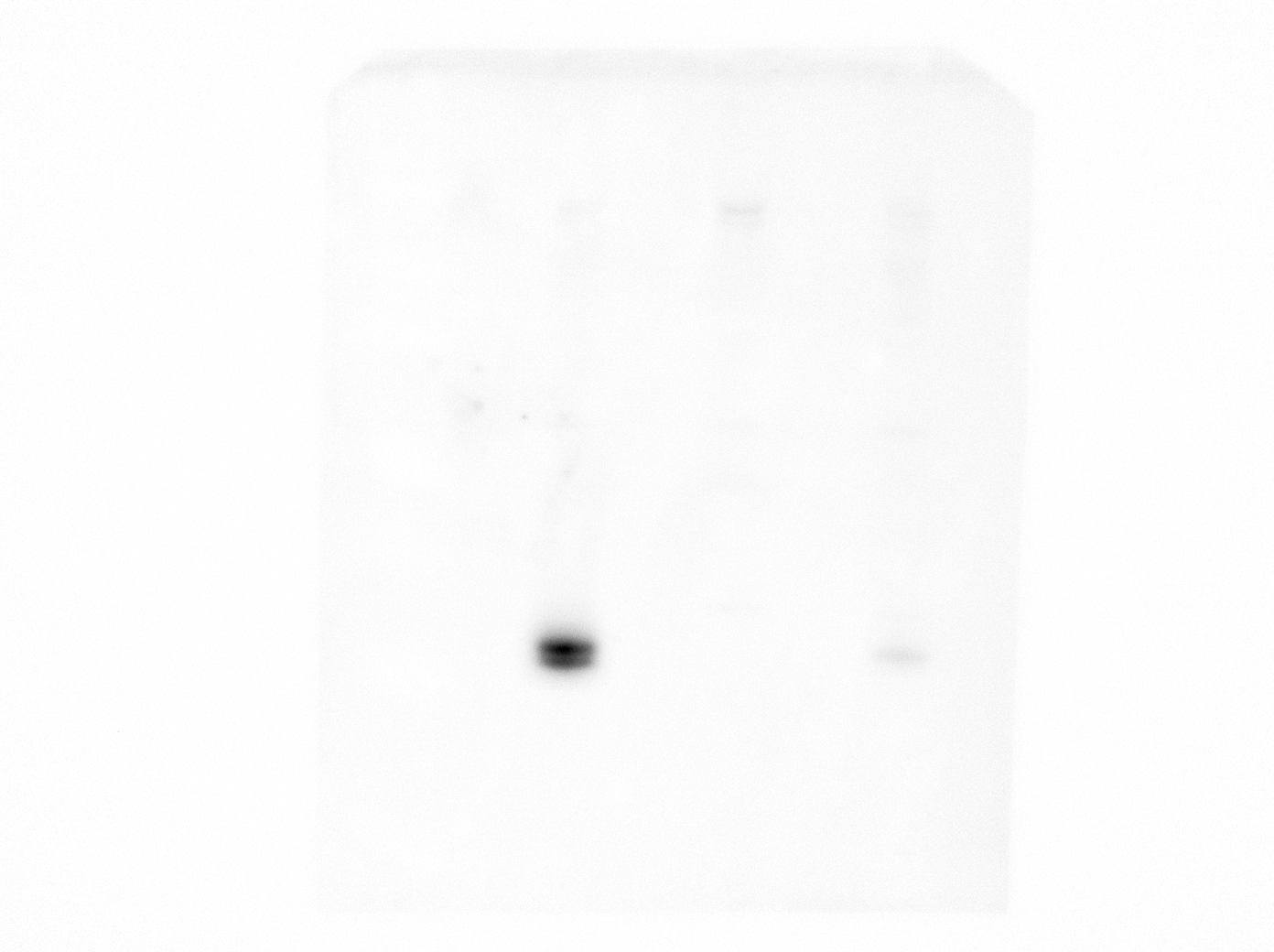

Supplement: Figure 5—source data 2. [file elife-105512-fig5-data2.zip › Figure 5 -source data 2/ax4_Set1_ash2_me2_10_secs copy.tif]

AX4 *casH*<sup>-</sup> *scd1*<sup>-</sup>

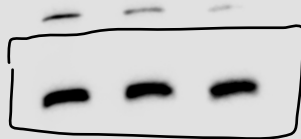

Supplement: Figure 6—source data 1. [file elife-105512-fig6-data1.zip › Figure 6 - source data 1/H3_hrp_30_secs.pdf]

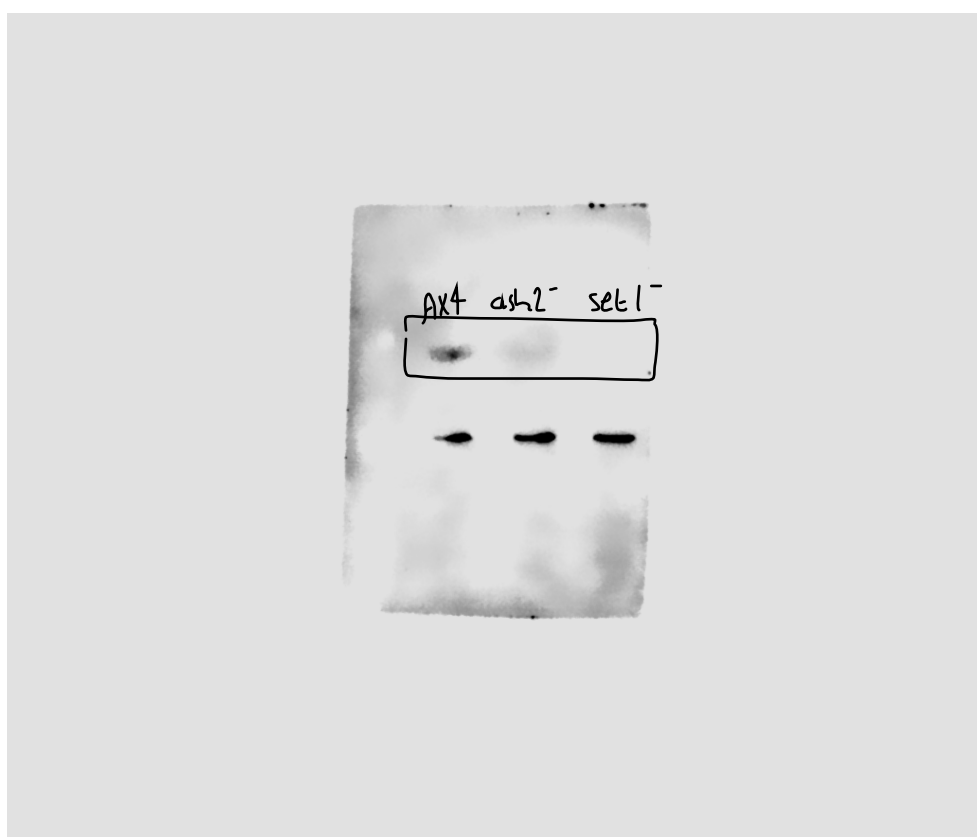

Supplement: Figure 6—source data 1. [file elife-105512-fig6-data1.zip › Figure 6 - source data 1/me1_hrp_5_mins.pdf]

A<sub>4</sub> ash2 set 1

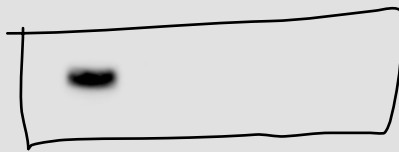

Supplement: Figure 6—source data 1. [file elife-105512-fig6-data1.zip › Figure 6 - source data 1/me3_hrp_30_secs.pdf]

AX4 ash2 sek1

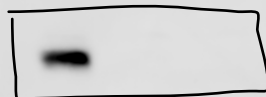

Supplement: Figure 6—source data 1. [file elife-105512-fig6-data1.zip › Figure 6 - source data 1/me2_hrp_5_mins.pdf]

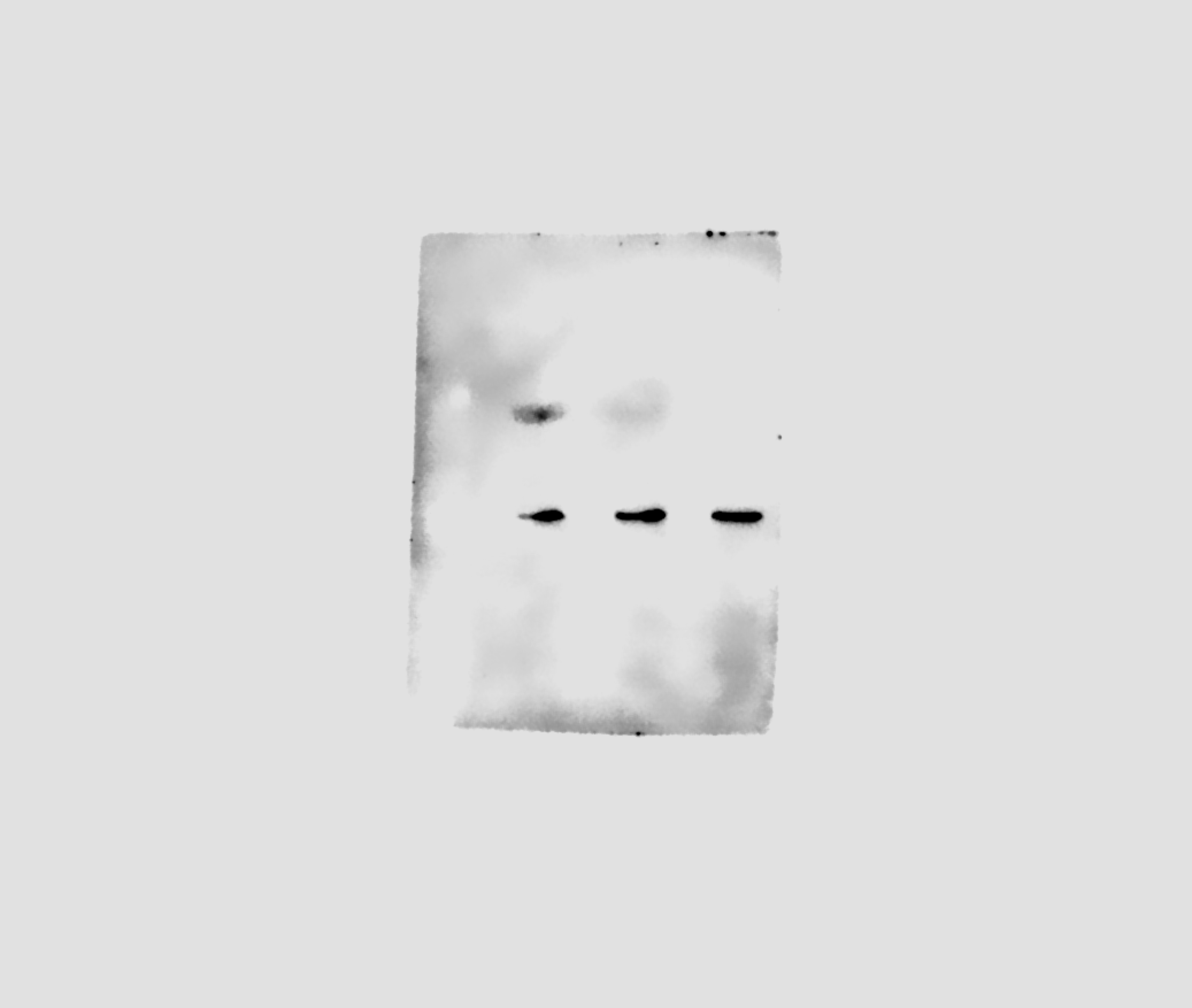

Supplement: Figure 6—source data 2. [file elife-105512-fig6-data2.zip › Figure 6 - source data 2/me1_hrp_5_mins copy.tif]

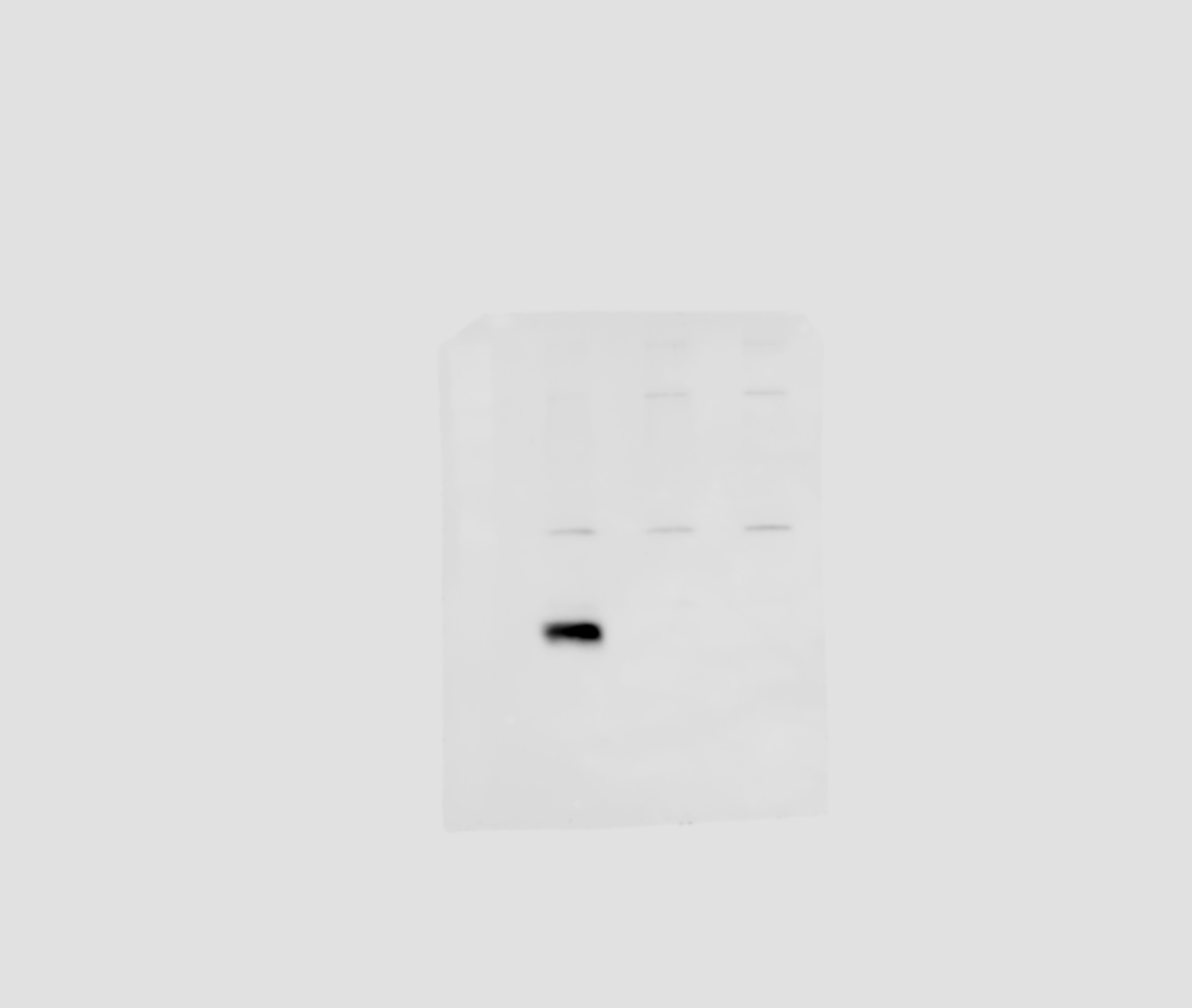

Supplement: Figure 6—source data 2. [file elife-105512-fig6-data2.zip › Figure 6 - source data 2/me2_hrp_5_mins copy.tif]

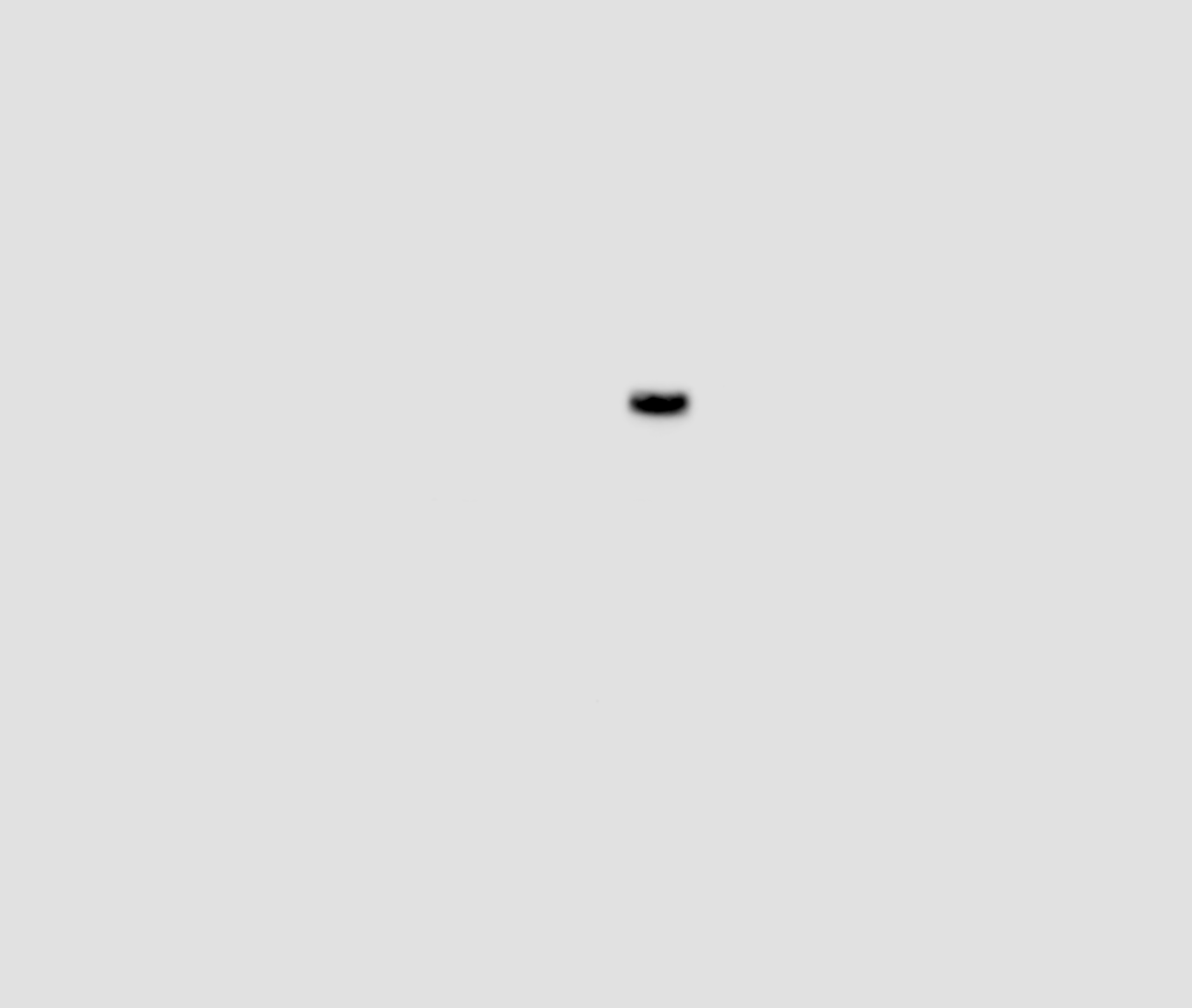

Supplement: Figure 6—source data 2. [file elife-105512-fig6-data2.zip › Figure 6 - source data 2/me3_hrp_30_secs copy.tif]

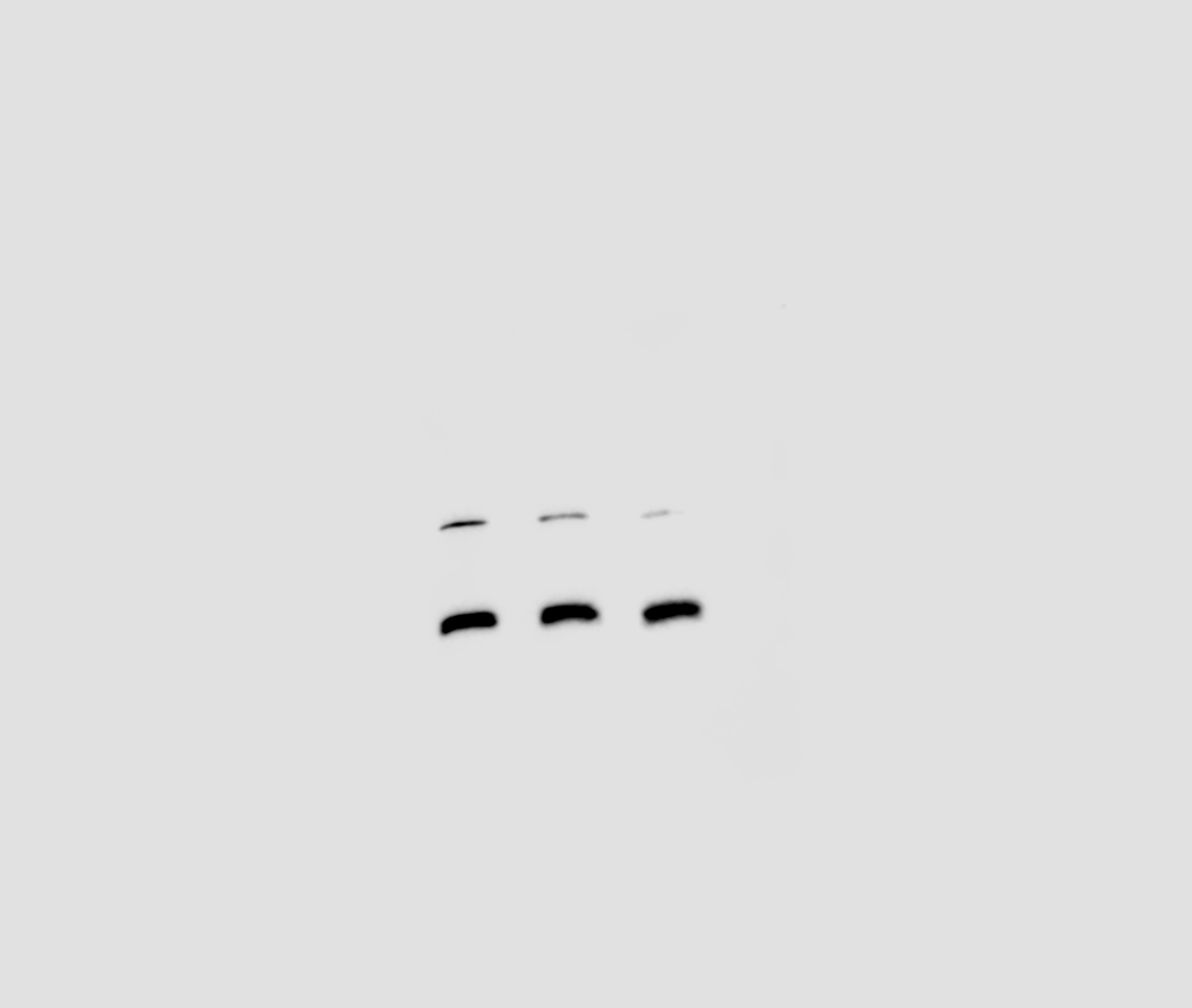

Supplement: Figure 6—source data 2. [file elife-105512-fig6-data2.zip › Figure 6 - source data 2/H3_hrp_30_secs copy.tif]

Ax4 set1 set1 flag

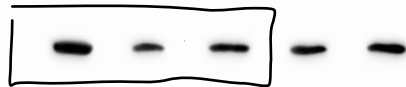

Supplement: Figure 6—figure supplement 3—source data 1. [file elife-105512-fig6-figsupp3-data1.zip › Figure 6 - source data 3/Actin_FLAG_invert.pdf]

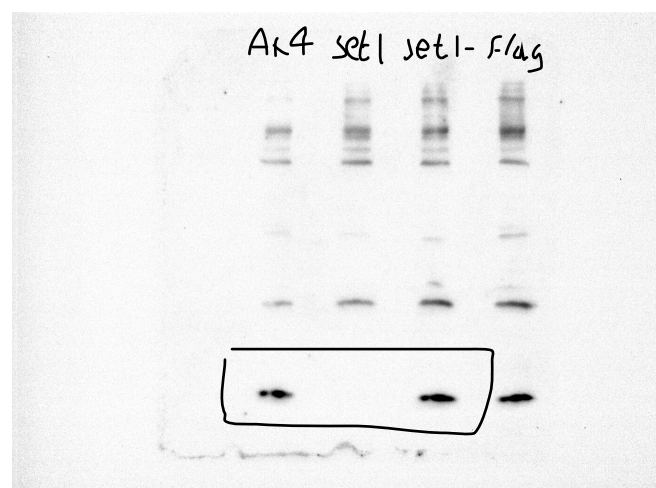

Supplement: Figure 6—figure supplement 3—source data 1. [file elife-105512-fig6-figsupp3-data1.zip › Figure 6 - source data 3/Me1_FLAG_invert.pdf]

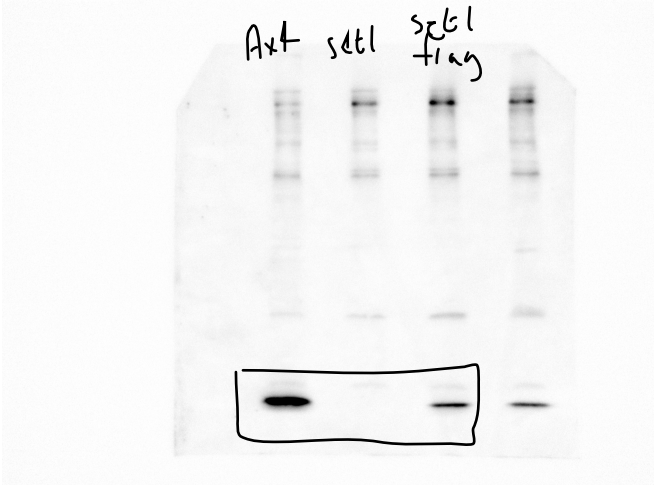

Supplement: Figure 6—figure supplement 3—source data 1. [file elife-105512-fig6-figsupp3-data1.zip › Figure 6 - source data 3/Me2_FLAG_invert.pdf]

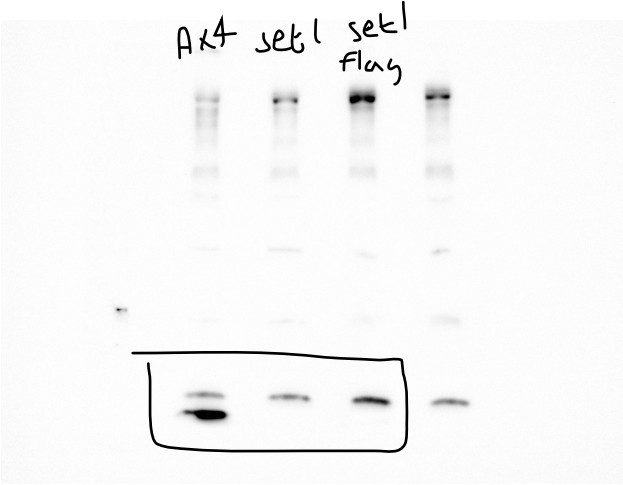

Supplement: Figure 6—figure supplement 3—source data 1. [file elife-105512-fig6-figsupp3-data1.zip › Figure 6 - source data 3/Me3_FLAG_invert.pdf]

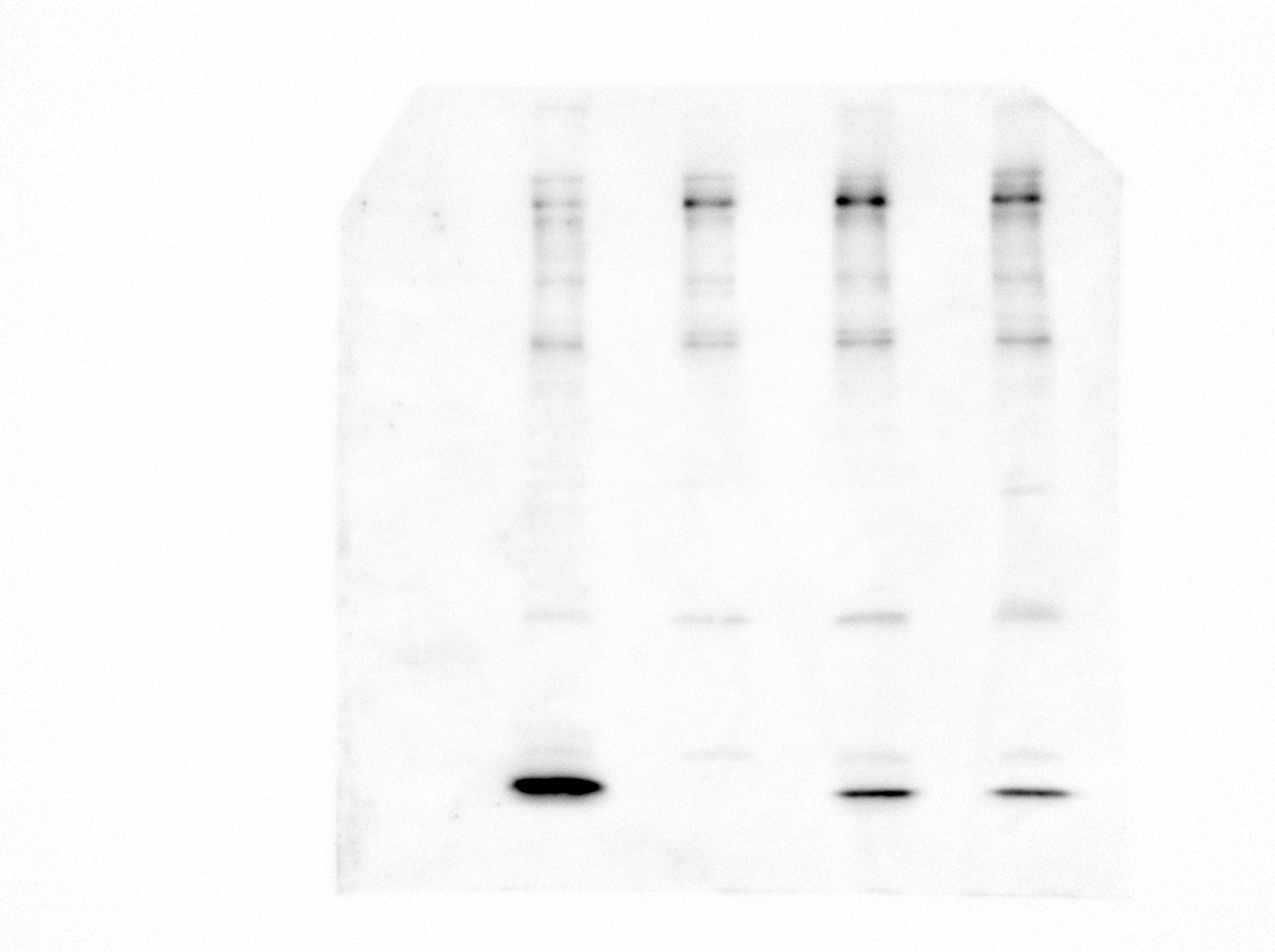

Supplement: Figure 6—figure supplement 3—source data 2. [file elife-105512-fig6-figsupp3-data2.zip › Figure 6 - source data 4/Me2_FLAG_invert copy.tif]

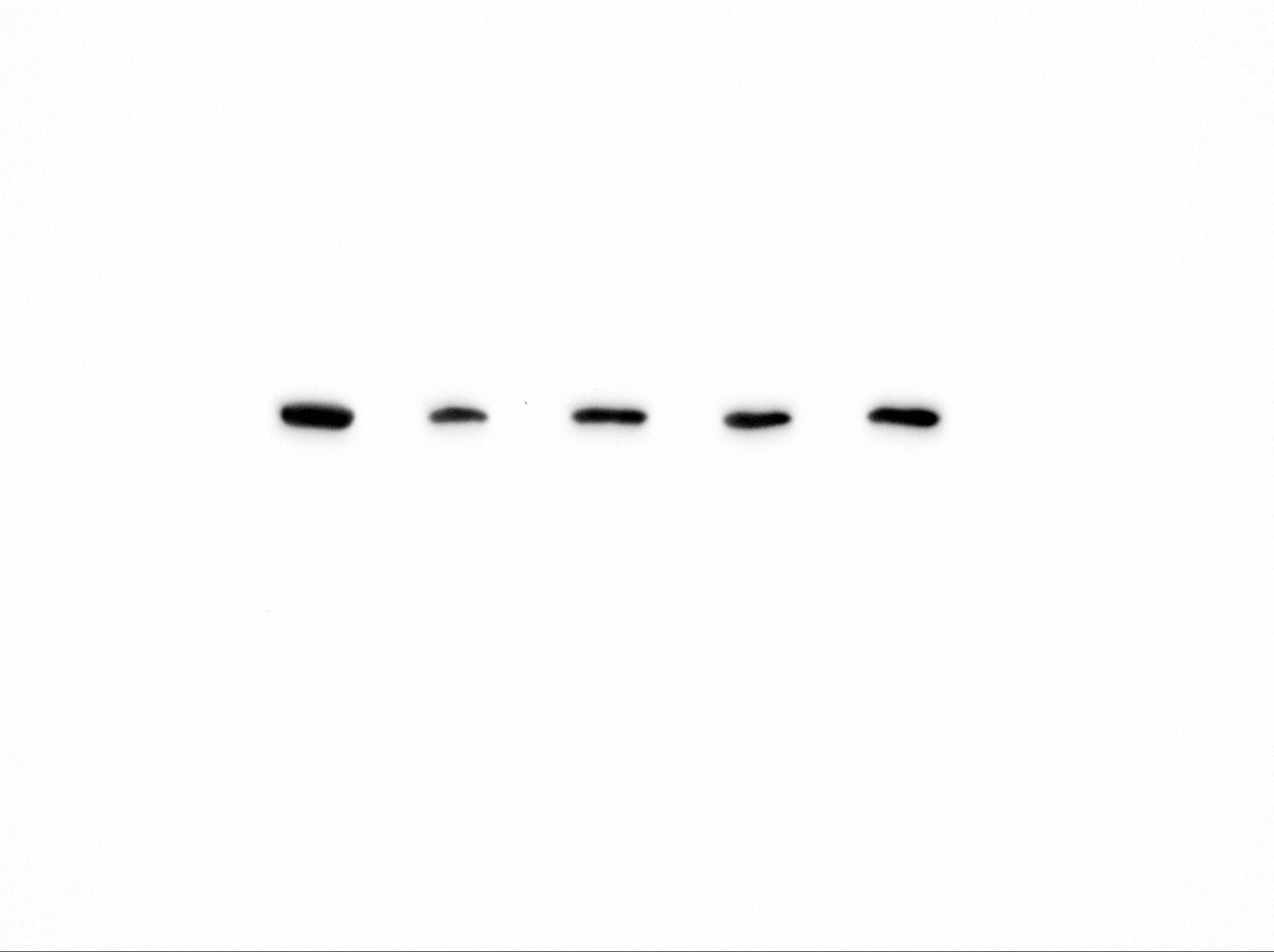

Supplement: Figure 6—figure supplement 3—source data 2. [file elife-105512-fig6-figsupp3-data2.zip › Figure 6 - source data 4/Actin_FLAG_invert copy.tif]

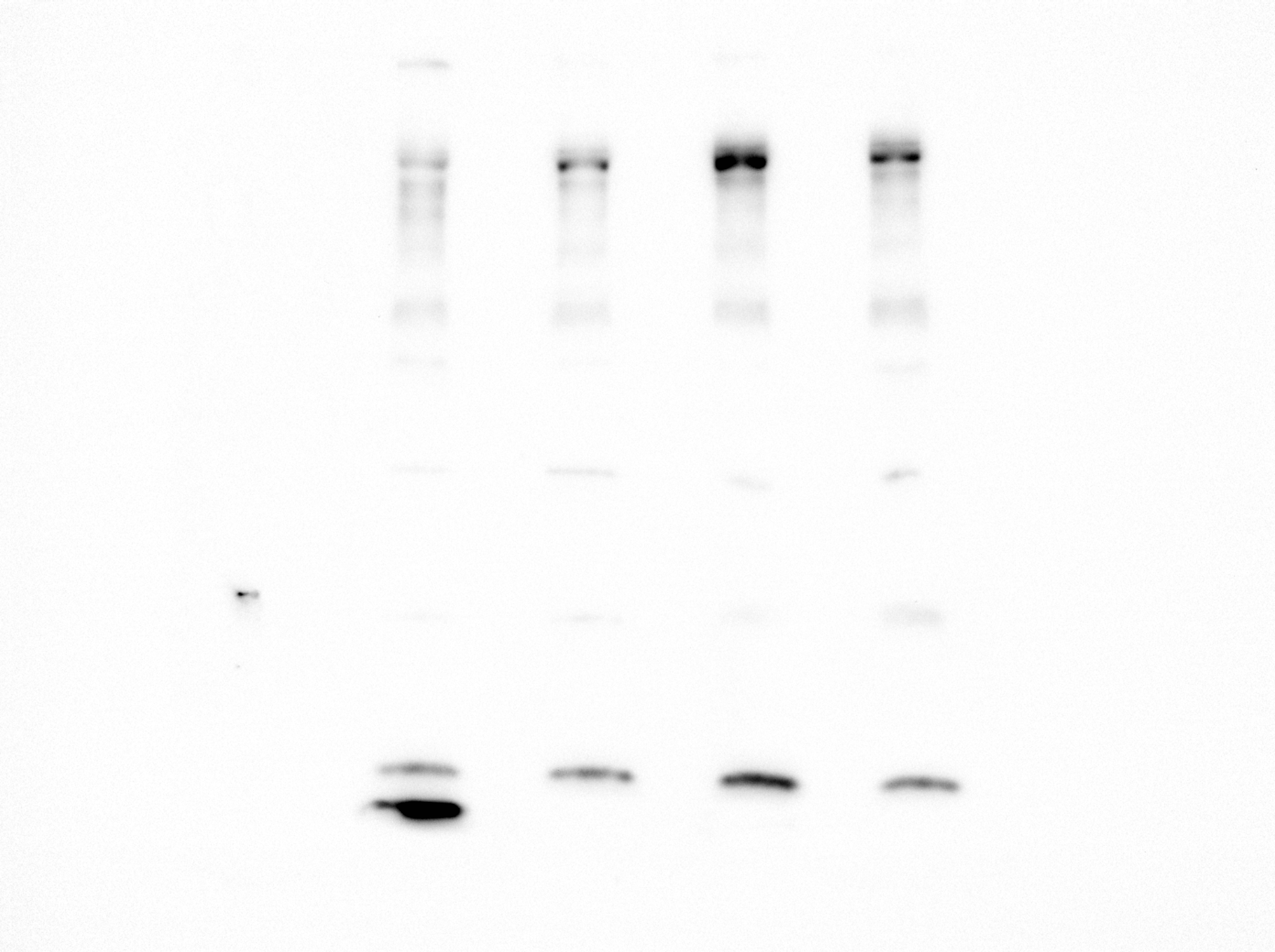

Supplement: Figure 6—figure supplement 3—source data 2. [file elife-105512-fig6-figsupp3-data2.zip › Figure 6 - source data 4/Me3_FLAG_invert copy.tif]

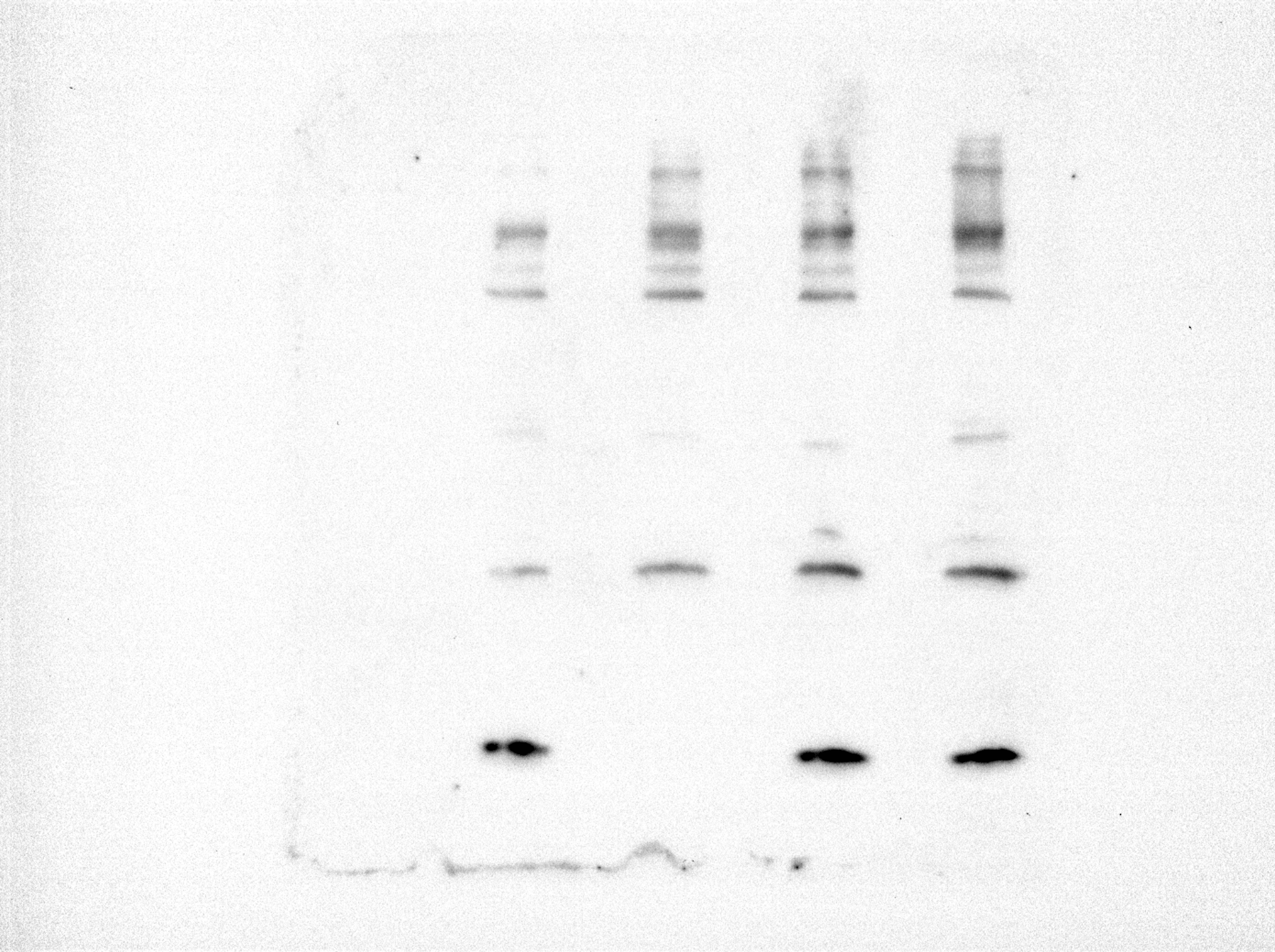

Supplement: Figure 6—figure supplement 3—source data 2. [file elife-105512-fig6-figsupp3-data2.zip › Figure 6 - source data 4/Me1_FLAG_invert copy.tif]
